# Opportunities for Ivory Nut Residue Valorization as a Source of Nanocellulose Colloidal Suspensions

**DOI:** 10.3390/gels9010032

**Published:** 2022-12-30

**Authors:** Enrique Javier Carvajal-Barriga, Jean-Luc Putaux, Pablo Martín-Ramos, Jennifer Simbaña, Patricia Portero-Barahona, Jesús Martín-Gil

**Affiliations:** 1 Neotropical Center for the Biomass Research, School of Biological Sciences, Pontificia Universidad Católica del Ecuador Av. 12 de Octubre 1076 y Roca, Quito 170523, Ecuador; 2 University Grenoble Alpes, CNRS, CERMAV, F-38000 Grenoble, France; 3 Instituto Universitario de Investigación en Ciencias Ambientales de Aragón (IUCA), EPS, Universidad de Zaragoza, Carretera de Cuarte s/n, 22071 Huesca, Spain; 4 Department of Agricultural and Forestry Engineering, ETSIIAA, Universidad de Valladolid, Avenida de Madrid 44, 34004 Palencia, Spain

**Keywords:** ivory nut, fractionation, nanocellulose, mannan, *Phytelephas aequatorialis*

## Abstract

Ivory nut seeds have been traditionally exploited in Central and South America for obtaining vegetable ivory. The residues from this industry are susceptible to valorization as a source of fatty acids (by organic extraction) and mannans (by alkaline dissolution and regeneration). Nonetheless, cellulose may also be recovered at the end of this fractionation process by acid hydrolysis and functionalization, with associated advantages over other lignocellulosic sources due to the absence of lignin in the endospermic tissue. In this work, various experimental parameters (sulfuric acid concentration, temperature, and hydrolysis time) were investigated to optimize the processing conditions for preparing stable nanocellulose suspensions after ultrasonication. The most stable nanocellulose gel (1 wt% solid content) was obtained after 4-h hydrolysis at 60 °C with 8 M H_2_SO_4_ and was characterized by using complementary tech-niques, including dynamic light scattering (DLS), transmission electron microscopy (TEM), X-ray powder diffraction (XRD), nano-fibril sulfation measurements, vibrational and solid-state nuclear magnetic resonance (CP/MAS 13C-NMR) spectroscopies, and thermal analysis. This nanocellulose hydrogel is susceptible to further utilization in various applications and fields, e.g., in agricul-ture for controlling the release of agrochemicals, in pharmaceutics for developing new dosage forms, and in the treatment of wastewater from the textile and paper industries.

## 1. Introduction

Sugarcane bagasse, cereal straw, wood chips, empty fruit bunches, and other agroindustrial leftovers are susceptible to valorization as renewable resources for obtaining chemicals, fuels, fertilizers, or materials. In the particular case of palm trees, some species are exploited as vegetable oil sources (e.g., *Elaeis guineensis* Jacq.), while other species-such as coconut trees and date palms- are grown for their edible fruits. Nonetheless, some of their by-products are also valuable. For instance, copra meal, obtained as part of the oil extraction process in coconut mills, is used as animal feed, given that it is rich in fibers, mannan, and proteins [1]. Taking into consideration that other seeds, such as those of avocado and grapes, have been successfully exploited in biorefineries to extract biobased materials and polymers [2,3], further investigation of the seeds and pits of some palm tree fruits as reservoirs of specific compounds susceptible of commercial exploitation is required.

This work focuses on the preparation of nanocellulose colloidal suspensions (prone to transition from solution-to-gel via crosslinking) from seeds of a palm species native to the Coastal Region of Ecuador, which grows in the tropical and subtropical habitats of the western flanks of the Andes chain: *Phytelephas aequatorialis* Spruce, also referred to as the ‘elephant palm tree’ [4]. The whitish and hard endosperm is used as a substitute for animal ivory [5,6] and also in the manufacturing of buttons for the fashion industry [5,7,8]. Such button manufacturing activity in Ecuador has declined due to the development of petroleum-based polymers but still generates over 400 t/month of endosperm residues, which at present are only used as fuel in industrial boilers [4]. Nonetheless, these residues, which feature typical moisture contents below 10 wt% [9], can be an interesting feedstock for the extraction of chemicals and materials with high added value due to their characteristic fractions (herein expressed in percent of dry matter): cell contents, mainly oils, 16 wt%; other cell contents such as proteins and other macromolecules, 5 wt%; mannan 72 wt%, and cellulose, 7 wt% [10,11,12,13]. In addition, the absence of lignin in the endosperm cell walls is particularly interesting for the extraction of cellulose nanofibrils, as it entails an advantage in terms of energy and time savings compared to wood-based nanocellulose [14], avoiding the use of polluting chemicals required for the removal of lignin [15]. 

Chanzy et al. reported on mannosyl polymers as the paramount energy source that accounts for more than 95% of the total sugars in the endosperm of ivory nuts [11]. Moreover, the crystalline structure of mannan was determined as mannan I (unit cell parameters a = 0.882 nm, b = 0.721 nm, and, in the fiber axis, c = 1.027 nm). In the case of the ivory nut, the main component of the endosperm is β(1,4)-linked-D-mannose [11].

Literature reports several approaches and protocols to extract and purify cellulose from sugar beet, citrus pulp, pears, apples, oil palm pulp, and other biomass sources for industrial applications [16,17,18,19,20]. However, the aforementioned feedstocks are from vegetal tissues different from the endosperm. Such differences among vegetal tissues, due to the structure of the cell walls [21], result in important differences in fractionation protocols. Hence, it is necessary to tailor *ad hoc* procedures adapted to the fractionation of endosperm tissues.

Nanocellulose materials can be used in a wide range of applications: as a nanofiller in polymer composites, in protective coatings, in membranes of filtration systems, in antimicrobial films, or as a substrate for electronic devices and batteries, to name but a few. They also have potential applications in agriculture (e.g., for the transport of bioactive products and pest control in crops), forestry (e.g., as sprayable carriers for the robust application of fire retardants in the prevention of forest fires), agroindustry (e.g., in the recovery of products from the food industry, for the maintenance of wine cellar pipes, or as food additives), and biomedicine (e.g., in tissue engineering, wound dressing and healing, drug delivery, 3D bioprinting, and biosensing) due to the low density, high tensile strength and low toxicity of nanocellulose [21,22,23,24,25,26,27,28,29]. In this research, we focused on the preparation of ivory nut nanocellulose colloidal suspensions that can be used in specific biotechnology applications for the creation of scaffolds and support matrices. Other nanocellulose suspensions, chiefly those obtained from abundant resources such as wood, are being utilized in large industries. 

Even though ivory nut possesses a hardness similar to that of mineral materials, it lacks lignin, which -as noted above- is a recalcitrant material that requires severe pretreatments to be removed from plant biomass. This chemical particularity of ivory nut would facilitate the fractionation of the compounds (i.e., mannan, cellulose, and extractives) and extraction of nanocellulose particles would be possible under milder conditions if compared to lignocellulosic feedstocks. Thus, the present work aims at evaluating a novel fractionation method built up exclusively for ivory nuts, putting together a specific sequence of steps that finally yield nanocellulose particles, fatty acids, and mannan. The fractionation method can be scaled up to pilot and industrial production. To confirm our hypothesis, we assessed the optimum operation conditions (in terms of sulfuric acid concentration, temperature, and reaction time) for the purification and functionalization of endospermic cellulose nanofibrils from ivory nut residues -after fatty acid and mannan extraction- to produce nanocellulose colloidal suspensions that may be valorized in industrial applications. The morphology, structure, and thermal properties of the extracted endospermic nanocellulose (ENC) were investigated using complementary characterization techniques.

## 2. Results and Discussion 

### 2.1. Fractionation from Ivory Nut Flour

**Scheme 1:**
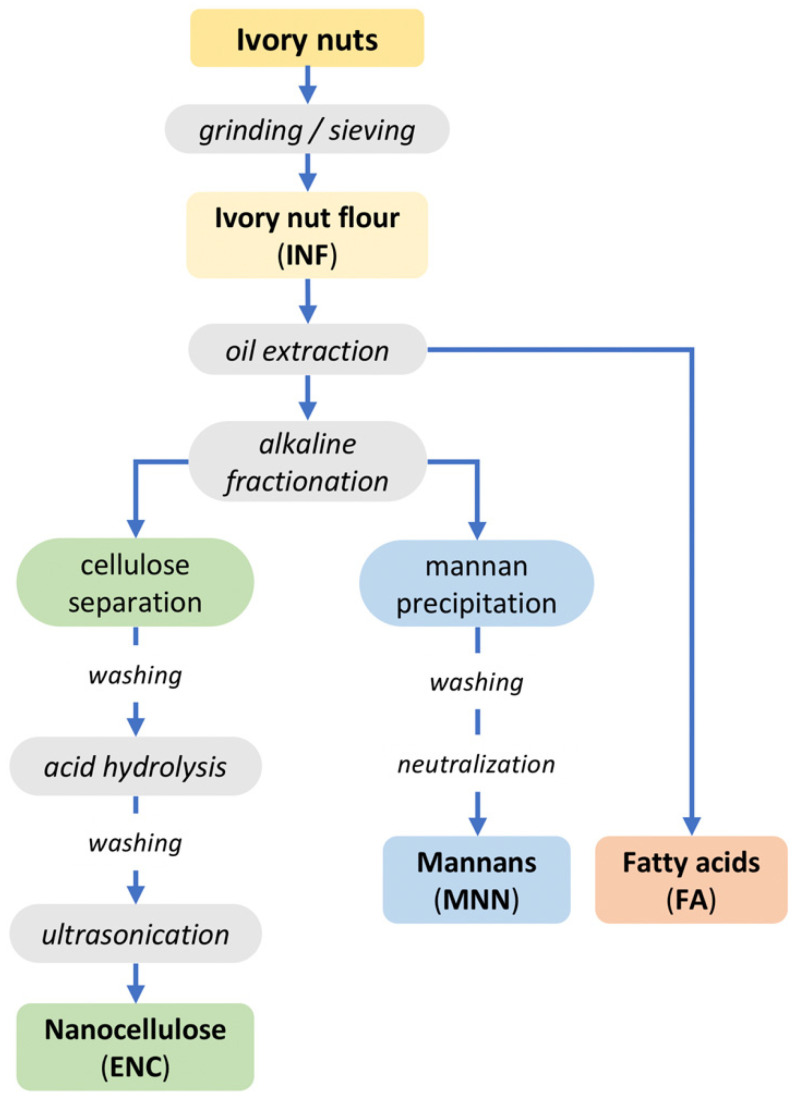
Ivory nut fractionation protocol.

Given that there is no systematic approach to the extraction of endosperm crude ivory nut parenchyma (ECINP) in the literature on the ivory nut, our inspiration was taken from wood pulping protocols. H_2_SO_4_ concentration, temperature, and reaction time were optimized to obtain clean suspensions. The method was based on a sequence of chemical treatments of the endosperm (Scheme 1). The oil was first extracted from the ivory nut flour and analyzed by gas chromatography. The saturated and unsaturated fatty acids constituting the oil were identified and quantified. The results are summarized in Supplementary Material Figure S1 and Table S1. The mannan fraction in the defatted flour was solubilized in alkali and regenerated in water. XRD and CP/MAS ^13^C-NMR analyses showed that the fraction was crystallized into the mannan I allomorph (Figures S2 and S3). The alkali-insoluble fraction from the defatted ivory nut flour was hydrolyzed with H_2_SO_4_, which yielded cellulose nanofibrils, sulfated by esterification of primary and secondary alcohol groups of the glucosyl units (by the sulfate groups from H_2_SO_4_). This method allowed separating mannans and ECINP cellulose with the concomitant sulfation of the cellulose nanofibrils. 

**Figure 1:**
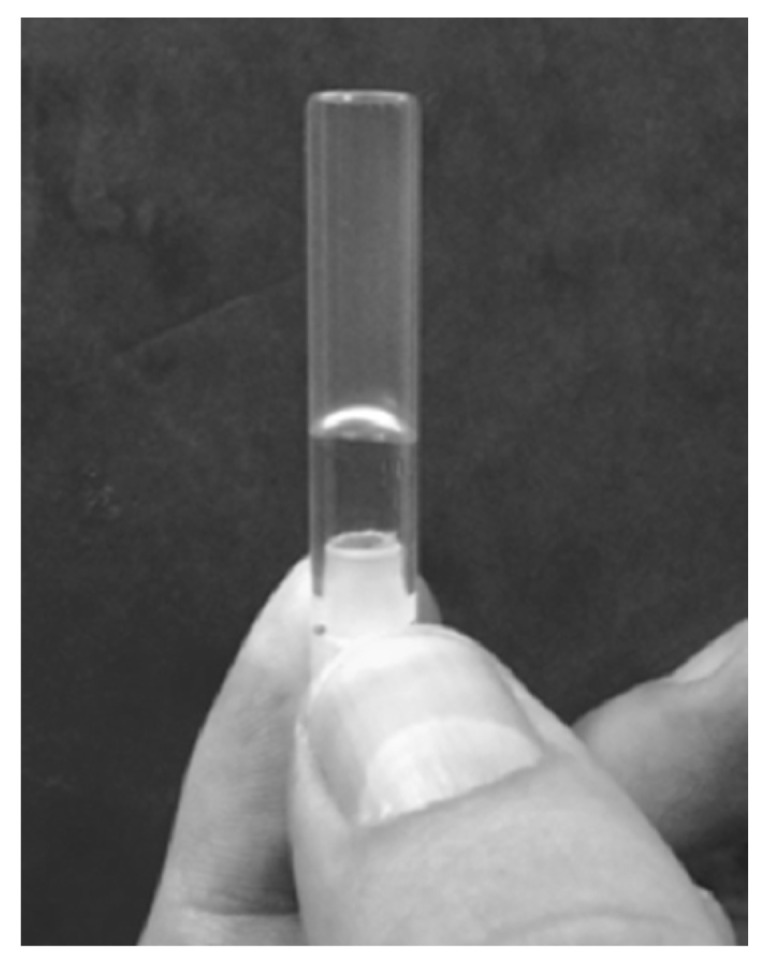
Photograph of the translucent and stable gel of ivory nut nanocellulose obtained from the endosperm of crude parenchyma treated with 8 M H_2_SO_4_ for 4 h at 30 °C and ultrasonicated (treatment 4 in Table 1).

Table 1 shows the results of the standardization protocol of ECINP fractionation. However, the D_h_ values listed in Table 1 are not realistic measurements of the particle size, given that cellulosic materials have a fibrillar nature and the D_h_ value calculated by DLS assumes that the particles are spheres with an equivalent diffusion coefficient. Hence, we used them as a relevant index (consistent with the qualitative turbidity of the suspensions) to compare the various samples. Treatments 3, 4, 5, 6, and 7 yielded mixtures with different degrees of turbidity. Upon comparison of samples 3, 4, and 7 (prepared with 8 M H_2_SO_4_) with samples 1, 2, 5, and 6 (produced with 4 M H_2_SO_4_), 8 M H_2_SO_4_ was selected as the more adequate concentration for ENC production. Treatments 1 and 2 yielded suspensions that were phase-separated due to incomplete hydrolysis. Reaction time also influenced the ECINP particle formation, as it could be observed for samples 5 and 6: the difference in homogeneity could be due to the reaction duration (1 vs. 4 h). Concerning the impact of temperature, for samples 1 and 2, the lower temperature (30 °C) produced two-phase mixtures, in contrast with test 5 (60 °C), which resulted in a more homogeneous product. There was a color change in sample 7, most likely because of the chemical transformation of sugars into furfural or hydroxymethylfurfural compounds. Sample 8, prepared under the most severe conditions (8 M H_2_SO_4_, 60 °C) was degraded and dissolved. In conclusion, treatment 4 yielded the product with the best characteristics: the suspension was homogeneous, translucent but slightly turbid, and exhibited a gel-like behavior at rest when the vial was turned around (Figure 1). Hence, this sample was selected for further characterization.

### 2.2. Characterization of the Fractions

#### 2.2.1. Nanocellulose Morphology

**Figure 2:**
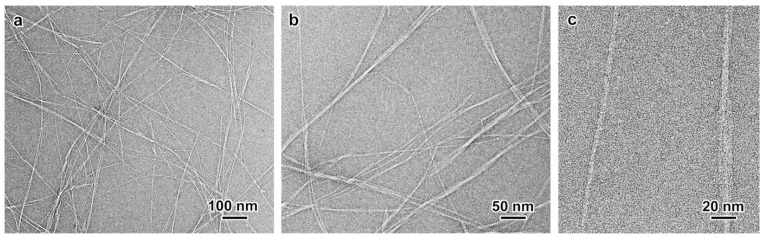
(**a**–**c**) TEM images of a negatively stained preparation of ivory nut nanocellulose extracted from the endosperm of crude parenchyma treated with 8 M H_2_SO_4_ for 4 h at 30 °C and ultrasonicated. The image in (**c**) shows an individual nanofibril (left) as well as a bundle of three (right).

TEM images of a negatively stained preparation of the dilute nanocellulose prepared by treatment 4 showed that the ultrasonication resulted in a fairly well-fibrillated specimen, with a few micrometer-long nanofibrils, individualized or still associated into bundles of a few units (Figure 2a,b). Higher magnification images allowed estimating an average nanofibril width of 3.5 nm (Figure 2c), in agreement with the typical values given for parenchymal nanocellulose [17,19]. 

#### 2.2.2. Structural Analyses

**Figure 3:**
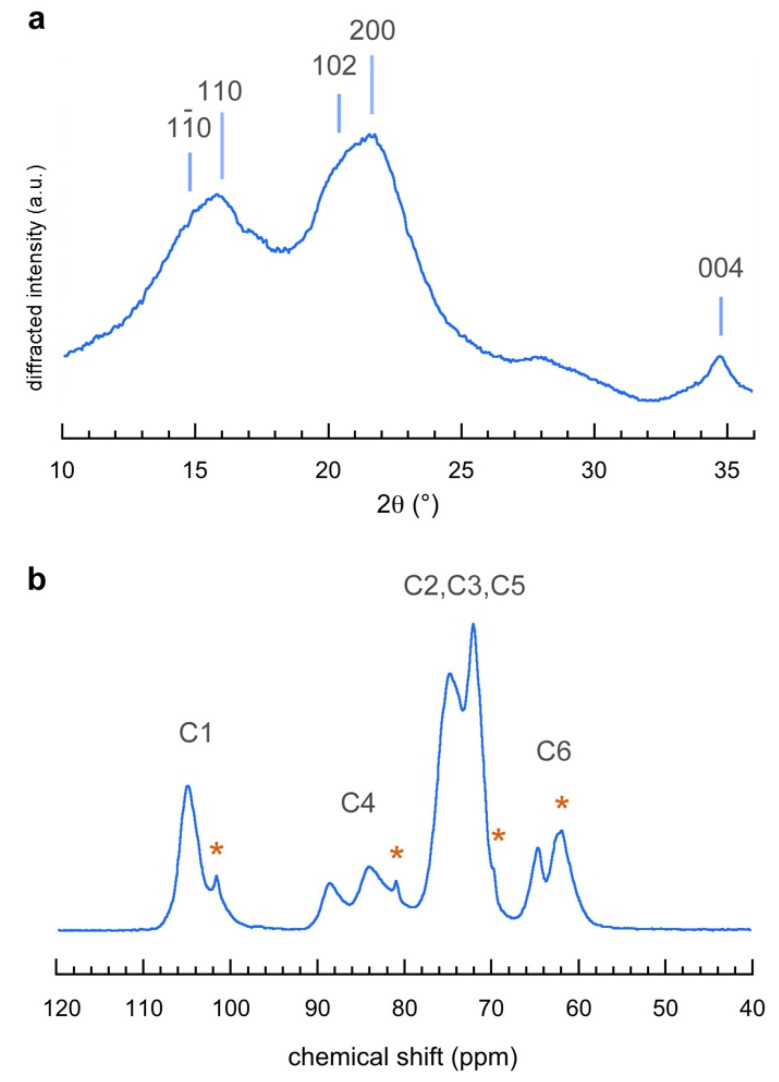
(**a**) X-ray powder diffraction pattern, and (**b**) CP/MAS ^13^C-NMR spectrum of the extracted nanocellulose (ENC). In (**a**), the Miller indices correspond to those of cellulose Iβ [33]. In (**b**), the asterisks indicate the presence of a minor fraction of residual mannan I in the ENC sample (Figure S3). * means other significant peaks.

The XRD pattern recorded from the ENC fraction (Figure 3a) was very similar to that of other parenchymal specimens [19,30,31,32]. Based on the Miller indexing of native cellulose Iβ [33], the lower-angle $1\bar{1}0$ and 110 reflections were merged into one another, forming a broader peak at about 15.8°. The higher broad peak at about 21.8° could be indexed as 200, with a contribution from the overlapping 102 reflection. Finally, the characteristic 004 reflection was located at 34.8°. Apart from the 004 peak, the shift of the broader XRD reflections with respect to those of cellulose I [34], previously reported for the parenchymal cellulose from blackberry (*Rubus fruticosus* L.) [35] and alkali-treated fibers of grapefruit (*Citrus grandis* L.) Osbeck) albedo [32], suggested that the structure of the ivory nut cellulose resembled that of cellulose IV_1_, a less ordered form of cellulose I [36]. 

The strongest signals in the CP/MAS ^13^C-NMR spectrum of ENC corresponded to those of cellulose Iβ and could be assigned to the carbon atoms of the glucosyl units (Figure 3b) with resonances at 104.9 ppm (C1), 88.6 and 84.0 ppm (C4), and 64.7 and 62.3 ppm (C4), in agreement with other spectra recorded from parenchymal cellulose [19,37]. The relative intensities of the two C4 peaks suggested a significant contribution of the surface chains of the nanofibrils (84.0 ppm) in comparison to that arising from chains in the crystalline core (88.6 ppm) [38], in line with the small width of the nanofibrils measured from the TEM images (Figure 2c). However, small peaks located at 101.6, 80.9, 69.8, and 61.9 ppm revealed that a minor fraction of mannan I was also present (Figure S3) [39,40]. 

#### 2.2.3. Vibrational Characterization of Endospermic Nanocellulose

**Figure 4:**
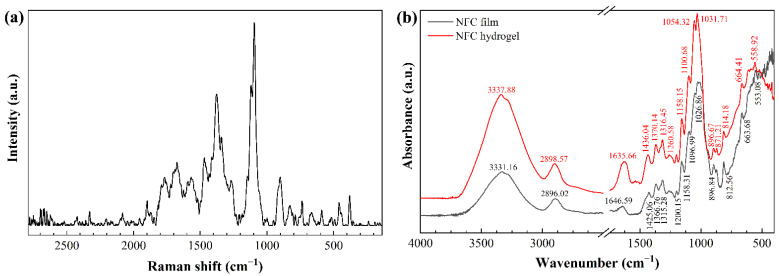
(**a**) Raman and (**b**) FTIR spectra of ivory nut nanocellulose (ENC).

The shifts in the Raman spectrum of ENC (Figure 4a) were in agreement with those of cellulose Iβ: 1470 cm^−1^ (CH_2_ and COH deformation), 1379 cm^−1^ (HCC, HCO, COH, and CH_2_ deformation), 1344 cm^−1^ (HCC, HCO bending, CH_2_ deformation, COH wagging), 1270 cm^−1^ (HCC, HCO bending, CH_2_ twisting), 1120 cm^−1^ (COC glycosidic linkage symmetric deformation, COC ring breathing), 1095 cm^−1^ (COC glycosidic link deformation, ring breathing symmetric stretching), 460 cm^−1^ (CCC, CCO ring deformation), and 380 cm^−1^ (CCC, CCO, CO ring deformation) [41]. 

The FTIR spectrum of ENC (Figure 4b) showed a strong band at 3334 ± 4 cm^−1^ in the region between 3700 and 3000 cm^−1^ associated with O-H stretching of intramolecular hydrogen bonds in cellulose I [30]. The band at 2897 ± 1 cm^−1^ corresponded to the presence of C–H stretching vibration and CH_2_–C(6)– bending vibration [42,43]. The band at 1641 ± 5 cm^−1^ could be attributed to the cellulose-water interaction, i.e., the -OH bending of absorbed water. The chemical structure of C–O–C pyranose ring skeletal vibration was observed at 1028 ± 2 cm^−1^ [44]. Furthermore, the peak at 1054 ± 4 cm^−1^ could be attributed to C–O and C–H stretching vibrations, which confirmed that cellulose occurred in its allomorph Iβ [45]. Finally, the peak at 896 cm^−1^ was attributed to the O–C–O stretching in C–H deformation of cellulose *β*-O-glucosidic linkage [46].

#### 2.2.4. Sulfation Degree of Endospermic Nanocellulose

**Figure 5:**
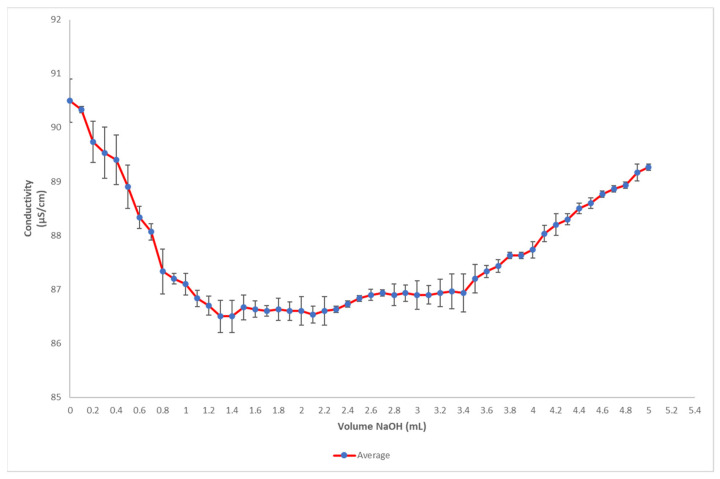
Titration curve of ivory nut nanocellulose from the endosperm of crude parenchyma treated with 8 M H_2_SO_4_ for 4 h at 30 °C.

The sulfation degree was calculated from the titration of nanocellulose suspensions extracted at 8 M H_2_SO_4_ for 4 h at 30 °C. The strong acid groups titration corresponds to sulfate groups esterified to the primary alcohol groups of cellulose fibers. The results are plotted in Figure 5. Using Equation (1) with *C_t_* = 2000 μM_NaOH_ L^−1^, *V_L_* = 0.0013 L, and *w* = 0.020075 g, the sulfation degree was calculated to be *X* = 129.51. The sulfation degree reported in the present work is lower than those previously reported in the literature. For instance, in the case of nanocrystals prepared by H_2_SO_4_ hydrolysis of cotton cellulose, the sulfation degree was 171 μM –SO_3_^−^ g^−1^ [47]. Other similar sulfation measurements reported in the literature were in the 173 to 214 μM –SO_3_^−^ g^−1^ range [48]. Other studies focused on the sulfation of nanocellulose crystals showed values fluctuating between 226.1 to 276.8 μM –SO_3_^−^ g_ENC_^−1^ [49]. According to the authors, considering the shape and size of these particular specimens, this sulfation degree means that about 30% of the available primary hydroxyl groups of cellulose nanocrystals are sulfated. If this data was comparable to ENC reported in the present work, it would mean that about one-fourth of the available primary hydroxyl groups of the nanofibrils would be sulfated. This explains the stability and homogeneity of the ENC suspension, as illustrated in Figure 1: the ionization of sulfate groups at the surface of nanofibrils provides negative charges that promote electrostatic repulsion and confer high colloidal stability to the ENC suspension. 

The pH of the suspension was 3.6 at a nanocellulose concentration of ca. 1% *w*/*v*. Moreover, the density was lower than that of water (*ρ* = 0.979 ± 0.02 g mL^−1^, *n* = 44), which may be explained by the generation of cavitation microbubbles occurring during low-frequency sonication and trapped into the consistent suspension. Due to this density, ENC tended to float on deionized water before it got dispersed. The cellulose content was 0.93 ± 0.11% *w*/*v* (*n* = 44). The resulting ENC suspension showed stability and transparency, as a result of the high separation capacity of nanoparticles in liquid media, which is a key factor in the formation of stable colloidal suspensions [50].

#### 2.2.5. Yield

The yields of cellulose obtained at the optimal conditions (8 M H_2_SO_4_ for 4 h at 30 °C) were:

- Yield_A_ obtained was 4.08%
- Yield_R_ obtained was 68.07%

#### 2.2.6. Thermal Analysis of Endospermic Nanocellulose

**Figure 6:**
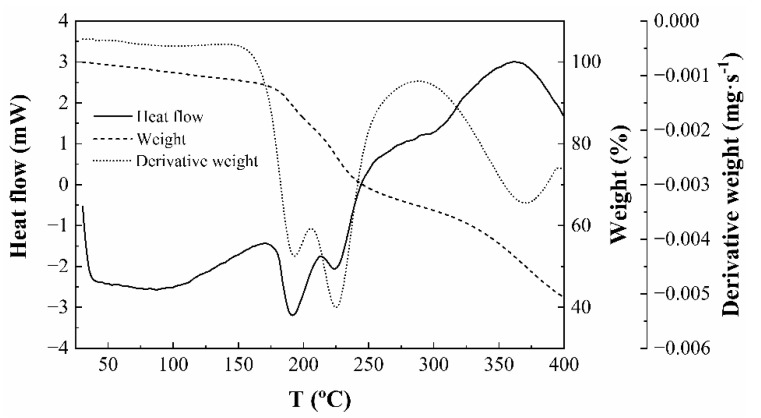
DSC (solid line, *y*-axis on the left side of the graph), TG (dashed line, first y-axis on the right side of the graph), and DTG (dotted line, second (rightmost) *y*-axis on the right side of the graph) curves of the ENC fraction.

Thermal stability is critical for the application of nanocellulose and thus, a pyrolysis kinetics analysis was conducted to determine the thermal degradation processes of the nanocellulose quantitatively. The thermal properties of the ENC fraction were characterized by thermogravimetric/derivative thermogravimetric (TG/DTG) and differential scanning calorimetry (DSC) analysis. The decomposition pattern of the ‘as obtained’ material is presented in Figure S4. The first thermal effects were a small weight loss at around 88 °C, related to dehydration, followed by another weight loss at 348 °C, between the temperatures reported by Cheng et al. [51] for cellulose nanocrystals prepared by acid hydrolysis (at 365.5 °C) and by Ketabchi et al. [52] for cellulose nanoparticles from kenaf fibers (at 335.2 °C). This degradation step represented the largest weight loss of the polymer and is usually considered a good indicator of the thermal stability of polymer composites [53]. The final decomposition was found to occur in two steps, with maxima at 454 and 720 °C, respectively. These results were similar to those reported for other nanocellulose TG analyses [54]. The thermal curves of cellulose gels and films (Figure 6) showed weight losses at 190 and 225 °C, followed by decomposition at 368 °C. These findings were in agreement with others described in the literature, except for the fact that the two effects below 230 °C appeared as a single effect at 219 °C in the work by Morais et al. [54], which may be explained by differences in heating rates. The reported thermal kinetics of the material should be useful with a view to a better design of polymer composite processing and to estimate the influence of the thermal decomposition of natural fibers on the composite properties.

### 2.3. Common Methods for Obtaining Nanocellulose and Nanocellulose Suspensions

Cellulose nanofibrils (CNFs) and cellulose nanocrystals (CNCs) can be obtained from different sources and different fibrillation methods [55]. In most cases, nanocellulose is prepared from cellulose isolated by acid hydrolysis or by degradation with enzymes, for example from sugarcane bagasse [56,57] or cotton [58,59], given that these procedures provide better yields than basic hydrolysis. For instance, CNC yields as high as 90.28% were reached by acid hydrolysis of rice straw when the reaction was conducted at a temperature of 30 °C and an acid concentration of 75 wt% for 5 h [60].

Other known methods of surface functionalization of nanocellulose particles provide ionic charges through various strategies such as phosphorylation, carboxymethylation, oxidation, and sulfonation. On the contrary, if hydrophobic interactions are required, they can be engineered through acetylation, etherification, silylation, urethanization, and amidation [61]. One of the most common derivatization methods is TEMPO-mediated oxidation, applied on highly pure cellulose resulting from an intensive process of woody materials into cellulose fibers. The present research went beyond the sole synthesis and functionalization of cellulose nanoparticles. We focused our hypothesis on the whole fractionation process which implies savings of time, reduced energy consumption, and environmental impacts due to the use of chemicals that are safe after simple neutralization processes. 

### 2.4. Applicability of the Obtained Nanocellulose Colloidal Suspensions

Biomedical applications of ENC suspensions are being developed at the moment with promising results. Indeed, the ivory nut ENC suspensions have shown their ability to crosslink into hydrogels through a controlled sol-to-gel transition mediated by the interaction with positive flanks of viral capsid proteins, and enhanced by Na^+^ ions. This crosslinking has been used to encapsulate viruses such as SARS-CoV-2 and HIV1 [62]. In this research, ENC colloidal suspensions were used for the high crosslinking capacity of the nanoparticles applied to the nanoencapsulation of viruses. This technology can be utilized to prevent viral infections. In another recent publication, ENC hydrogels were reported for their use in the formation of surface-activated spheroids to capture Cr VI for water remediation [63]. 

Both examples of innovative uses of ENC colloidal suspensions revealed that their utilization goes beyond their applications in the design of high-performance materials (i.e., materials for reinforcement or coating of surfaces) where specific physical-mechanical characteristics are crucial for the design of endurable materials. Regarding the applicability of ENC colloidal suspensions based on their characteristics, analysis of strength, strain, or rheological characterizations yield less information than analysis for the determination of cellulose type, surface activation with SO_3_^−^ and the shape and size of the nanoparticles.

Another application of the ENC suspensions reported in the same study of the antiviral activity of ENC, reported by Carvajal Barriga et al. [62], shows the crosslinked matrix yields scaffolds that can be used in 3D cell cultures, bringing procedural advantages in biotechnology laboratories. 

## 3. Conclusions

The absence of lignin in ivory nut endosperm offers new opportunities for the valorization of the leftovers of the button manufacturing industry beyond fatty acids and mannans recovery, given that highly pure and homogeneous nanocellulose can be obtained from it. Ivory nut is not a rich source of PNC to be exploited to attain exclusively that source, since there are other sources that contain much more cellulose, like wood pulp, nevertheless, the shortened process due to the lack of lignin, presents quite noticeable advantages. In addition, the yield obtained (68.08 wt%) of the existing cellulose in the ivory nut, is a high value if compared to other sources that yield between 20 to 75% (wt%).

The analysis of purification and functionalization conditions allowed to select the treatment with 8 M H_2_SO_4_ for 4 h at 30 °C, followed by ultrasonication, as the most suitable process parameters, given that it yielded a homogeneous, translucent, and stable hydrogel with a cellulose content of 0.93 ± 0.11% *w*/*v*. XRD results allowed the classification of the pure cellulose nanofibrils as cellulose type IV_1_, with stabilizing charged sulfate groups at their surface. The sulfation degree was calculated to be 130 μM –SO^3−^ g^−1^ which is relatively low if compared to other sulfated nanoparticles reported in the literature. Nonetheless, the sulfation degree suffices to keep the colloidal stability of the suspension. The ionization of these sulfate groups at the surface of nanofibrils provided negative charges that promoted electrostatic repulsion and conferred high colloidal stability to the ENC suspension. The presented *ad hoc* procedure, specifically designed for the fractionation of endosperm tissues, is susceptible to be scaled up to yield highly pure nanocellulose from ivory nut, and features advantages in terms of energy and time saving compared to processes based on lignocellulosic feedstocks, while avoiding the use of polluting chemicals required for the removal of lignin.

## 4. Material and Methods

### 4.1. Feedstock

Ivory nut leftovers were obtained from a button factory in Manabí province, Ecuador. The hard and woody endocarp was completely removed by polishing to expose the endosperm. The seeds were cracked with a hammer and small pieces were ground by ball-milling. The ivory nut flour (INF) was screened in a 100-μm mesh sieve and utilized in further fractionation processes, as depicted in Scheme 1. The experimental design and methods utilized in those processes are detailed in the patent application IEPI-2016-61010 [64].

The cellulose pellet obtained after the oil extraction and alkaline fractionation steps was washed with deionized water until reaching pH 8. Subsequently, eight treatments were carried out (Table 1), at two working temperatures (30 °C for treatments 1 to 4, and 60 °C for treatments 5 to 8) and with reaction times of either 1 or 4 h. For each treatment, the cellulose pellet was resuspended in 27.5 mL of deionized water in a reaction flask kept at 4 °C. Concentrated H_2_SO_4_ (4 or 8 M, 22.5 mL) was then added dropwise to the flask at a constant flow rate of 1 mL min^−1^. The reaction was kept at 30 °C in a cold-water bath for one hour (treatments 1, 3, 5, and 7) or four hours (treatments 2, 4, 6, and 8), with continuous stirring at 300 rpm.

Finally, the resulting suspension was centrifuged at 4200 rpm for 10 min and the supernatant was discarded. The pellet was washed with deionized water in a centrifuge at 4200 rpm for 10 min to reach a pH of 4. The resulting endospermic nanocellulose sample will be referred to as ‘ENC’. The resuspended pellet was subjected to continuous sonication with a Branson Ultrasonics (Danbury, CT, USA) S-250A sonicator in an ice bath for 3 min at 20 kHz. The resulting suspension was filtered using a vacuum pump with a 35-μm pore size cloth filter and stored at 4 °C for further analysis. Validation studies were conducted in triplicate. 

### 4.2. Characterization

#### 4.2.1. Particle Size Measurement

The suspensions were characterized by dynamic light scattering (DLS). The suspensions were diluted to 0.001 wt% and analyzed in terms of hydrodynamic diameter (D_h_) and polydispersity index (PDI) using a VASCO particle size analyzer (Cordouan Technologies, Pessac, France) at 25 °C.

#### 4.2.2. Transmission Electron Microscopy (TEM)

Droplets of 0.001 wt% aqueous suspensions were deposited onto glow-discharged carbon-coated TEM grids. Before drying, the preparation was negatively stained with 2% uranyl acetate. The specimens were observed using a JEOL JEM-2100-Plus microscope (Akishima, Tokyo, Japan) operating at 200 kV. The images were recorded with a Gatan Rio 16 digital camera (Ametek Inc., Devon-Berwyn, PA, USA). 

#### 4.2.3. X-Ray Powder Diffraction (XRD)

Drops of nanocellulose suspension were allowed to dry on a Teflon surface and the resulting film fragments were poured into capillaries. The capillaries were flame-sealed and X-rayed under vacuum, in transmission mode, using a Philips PW3830 generator (Amsterdam, The Netherlands) operating at 30 kV and 20 mA (Ni-filtered CuKα radiation, wavelength λ = 0.1542 nm). Two-dimensional diffraction patterns were recorded on Fujifilm (Tokyo, Japan) imaging plates and read using a Fujifilm BAS 1800 II bio-imaging analyzer. Diffraction profiles were calculated by rotationally averaging the ring patterns. 

#### 4.2.4. Cellulose Nanofibril Sulfation Measurement 

Due to the sulfuric acid treatment, the cellulose nanofibrils were esterified with –SO^3−^ groups. Their concentration was measured by conductometric titration using a Mettler Toledo SevenGo SG pH meter (Columbus, OH, USA). The sample was prepared as follows: 2.5 g of an 0.8 wt% aqueous nanocellulose suspension was diluted in 120 mL in a 1 mM NaCl solution and neutralized by adding dilute NaOH (2 mM). The titrant (NaOH) was added in aliquots of 0.1 mL at 30-s intervals [65]. Finally, the sulfate group content was determined after three subsequent measurements [66]. The average value of the volume of NaOH and conductometry were taken to plot the titration curve. The ester sulfate content *X* (in μmol g^−1^) of the samples was calculated using Equation (1):

(1) $$X=\frac{C_{t}\times V_{L}}{w}$$

where C_t_ is the concentration of the NaOH solution, in μmol L^−1^, V_L_ is the volume in L of NaOH solution consumed at the first intersection point, and w is the oven-dried weight of the sample expressed in g.

#### 4.2.5. Yield

The yield was measured by triplicate, by drying 5 mL of the colloidal suspensions for 72 h at 80 °C in a lab oven (Memmert model 500). There are two kinds of yield calculations that were named Yield_A_ and Yield_R_. The first one refers to the wt% of cellulose recovered from the initial biomass input in dry weight; the second one refers to the wt% of cellulose recovered and compared to the theoretical values of cellulose content in ivory nut provided by previous works [13,67,68]. The moisture content of the material was previously calculated as 7 wt%. Thus, we introduced a 0.93 correction factor in the Yield_A_ equation. Both equations employed were adapted to the following general Equation (2):

(2) Yield (wt%)=(dry weight of biomass recovered/dry weight of total biomass)×100

Thus, Equation (3) is as follows:

(3) $${\mathrm{Yield}}_{A}\;\left(\mathrm{wt}\%\right)=0.93\times \left(\mathrm{dry}\;\mathrm{weight}\;\mathrm{of}\;\mathrm{cellulose}\;\mathrm{recovered}/\mathrm{total}\;\mathrm{feedstock}\;\mathrm{weigh}\right)\times 100$$

and Equation (4) is:

(4) $${\mathrm{Yield}}_{R}\;\left(\mathrm{wt}\%\right)=\left(\mathrm{dry}\;\mathrm{weight}\;\mathrm{of}\;\mathrm{biomass}\;\mathrm{recovered}/\mathrm{total}\;\mathrm{feedstock}\;\mathrm{weight}\times 0.07\right)\times 100$$

#### 4.2.6. Vibrational Spectroscopy

Raman spectra were acquired at room temperature in the 138-2785 cm^−1^ range at 1 cm^−1^ spectral resolution on a Jasco (Easton, MD, USA) NRS-5100 dispersive Raman system (632.78 nm laser line; 600 lines mm^−1^ dispersion grating; 50 × 1000 µm slit; rejection filter 632.8 nm; resolution 4.92 cm^−1^, 2.59 cm^−1^ pixel^−1^; objective lens MPLFLN 20×; laser power 1.7 mW; attenuator open; 4-stage Peltier-cooled CCD (UV-NIR range, 1024 × 255 pixels)). 

The Fourier-transform infrared (FTIR) spectrum was characterized using a Thermo Scientific (Waltham, MA, USA) Nicolet iS50 spectrometer, equipped with an in-built diamond attenuated total reflection (ATR) system. The spectra were collected in the 400–4000 cm^−1^ region at room temperature, with a 0.5 cm^−1^ spectral resolution. Sixty-four scans were accumulated and the resulting interferograms were averaged.

#### 4.2.7. CP/MAS ^13^C-NMR Spectroscopy

The samples were packed into zirconia rotors and analyzed with a Bruker (Billerica, MA, USA) Avance III 400 MHz spectrometer (^13^C frequency of 100.6 MHz) using cross-polarization (CP) and magic angle spinning (MAS), at a 12 kHz spinning speed, a sweep width of 29761 Hz, and a recycle delay at 2 s. Each spectrum was averaged over 6000 scans. The ^13^C chemical shifts were calibrated with the resonance of the glycine carboxyl group at 176.03 ppm. 

#### 4.2.8. Thermal Analysis 

The thermogravimetric/derivative thermogravimetric (TG/DTG) and differential scanning calorimetry (DSC) analyses of the ENC suspensions were conducted with a Perkin-Elmer (Waltham, MA, USA) STA6000 simultaneous thermal analyzer by heating the sample (4.59 mg) in a slow stream of N_2_ (20 mL min^−1^) from room temperature up to 400 °C, at a heating rate of 20 °C min^−1^. The data analysis was performed using the Perkin Elmer Pyris v.11 software. Additional TG/DTG measurements up to 900 °C were carried out with a TA Instruments (New Castle, DE, USA) Q-500 apparatus, at a heating rate of 10 °C min^−1^, under N_2_ flow (20 mL min^−1^). 

#### 4.2.9. Density and Cellulose Content in ENC Suspensions

A volume of 7.5 mL of suspensions was first weighed (net weight) to determine the density and then dried at 50 °C for 8 h to form a dry nanocellulose membrane. The cellulose content (wt%) was estimated according to Equation (5):

(5) $$\%\;\mathrm{cellulose}\;=\frac{\mathrm{dry}\;\mathrm{weight}}{\mathrm{net}\;\mathrm{weight}}\times 100$$

## Figures and Tables

**Table 1:** Experimental conditions of the tests performed to optimize the extraction method from endosperm crude ivory nut parenchyma, together with the physical characteristics of the resulting samples. Particle size (D_h_) and polydispersity index (PDI) were measured after ultrasonication of the samples.

Treatment	H_2_SO_4_ (M)	T (°C)	Time (h)	Consistency	Color	Turbidity	Number of Phases	D_h_ (nm)	PDI
1	4	30	1	watery	translucent	no	2	828	1.16
2	4	30	4	watery	translucent	no	2	839	1.34
3	8	30	1	gel	hazy	low	1	584	0.24
4	8	30	4	gel	translucent	very low	1	398	0.13
5	4	60	1	gel	white	high	1	483	0.18
6	4	60	4	gel	white	high	1	312	0.25
7	8	60	1	gel	red	high	1	475	0.09
8	8	60	4	watery	dark	no	1	n/a	n/a

## Data Availability

The data presented in this study are available on request from the corresponding author.

## Supplementary Materials

The following supporting information can be downloaded at: https://www.mdpi.com/article/10.3390/gels9010032/s1, Figure S1: Seed oil fatty acid profile; Table S1: Fatty acid content; Figure S2: XRD profiles of ivory nut flour and recrystallized mannans; Figure S3: CP/MAS ^13^C-NMR spectra of ivory nut flour and recrystallized mannans; Figure S4: Thermogravimetric profiles of ENC.
