# Supplementary material for: Opportunities for Ivory Nut Residue Valorization as a Source of Nanocellulose Colloidal Suspensions
Source: Gels. 2022 Dec 30;9(1):32. doi: 10.3390/gels9010032 (PMC9857895; doi:10.3390/gels9010032)
Supplement: Supplementary file 1 [file gels-09-00032-s001.zip › gels-2078378-supplementary.pdf]

Supplementary Material

Opportunities for Ivory Nut Residue Valorization as a Source of Nanocellulose Colloidal Suspensions

Enrique Javier Carvajal-Barriga [1,*], Jean-Luc Putaux [2], Pablo Martín-Ramos [3,4], Jennifer Simbaña [1], Patricia Portero-Barahona [1] and Jesús Martín-Gil [4]

1 Neotropical Center for the Biomass Research, School of Biological Sciences, Pontificia Universidad Católica del Ecuador Av. 12 de Octubre 1076 y Roca, 170523 Quito, Ecuador
2 Univ. Grenoble Alpes, CNRS, CERMAV, F-38000 Grenoble, France
3 Instituto Universitario de Investigación en Ciencias Ambientales de Aragón (IUCA), EPS, Universidad de Zaragoza, Carretera de Cuarte s/n, 22071 Huesca, Spain
4 Department of Agricultural and Forestry Engineering, ETSIIAA, Universidad de Valladolid, Avenida de Madrid 44, 34004 Palencia, Spain
* Correspondence: ejcarvajal@puce.edu.ec

1.1. Seed oil fatty acid profile

The FA profile of the seed oil was determined by gas chromatography with flame ionization detector (GC-FID) according to the AOAC 996.06 method (AOAC, 2005) by using an Agilent 7890A gas chromatograph with a flame ionization detector, with a Agilent HP-88; 60 m × 250 µm × 0.2 µm column, with helium at a flow of 1.5 mL min^{-1}. The initial temperature was 125 °C for 2 min, with a 6 °C min^{-1} increment up to 145 °C, keeping it for 25 min, an increment of 2 °C min^{-1} up to 220 °C and holding it for 15 min (total time: 83 min). Detector at 260 °C. Injector at 250 °C with a 70:1 division ratio. Software: 250 °C with a 70:1 division ratio. Standard: Supelco FAME mix C4-C24.

P. aequatorialis seeds contain a high amount of saturated and monounsaturated fatty acids accounting for 96 wt% of the total fatty acids composition (**Figure S1**). Oleic and palmitic acids represent about 86% of the content, while lauric, myristic, stearic and linoleic acids occur in smaller amounts (**Table S1**). Compared to other palm tree seeds, for example *Phoenix dactylifera,* ivory nut contains less lauric acid, while *P. aequatorialis* contains more palmitic acid (Hamza et al., 2021).

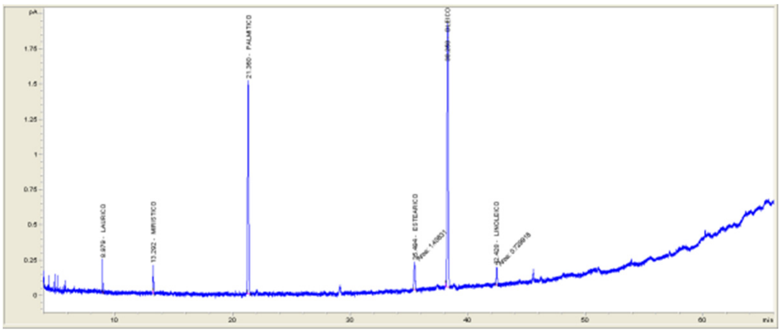

Figure S1. Gas chromatogram of *P. aequatorialis* fatty acid methyl esters from the oil retrieved by Soxhlet extraction.

Table S1. Fatty acid content in the *Phytelephas aequatorialis* species.

Fatty acid	wt%
Lauric	2.10
Myristic	2.77
Palmitic	36.88
Stearic	5.45
Oleic	49.98
Linoleic	2.83

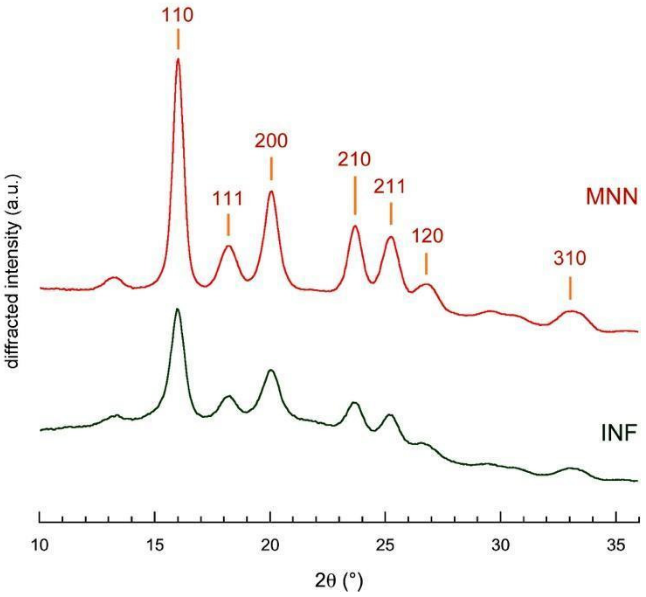

Figure S2. XRD profile of ivory nut flour (INF) and recrystallized mannans (MNN). The Miller indices correspond to those of mannan I (Hori et al., 2007).

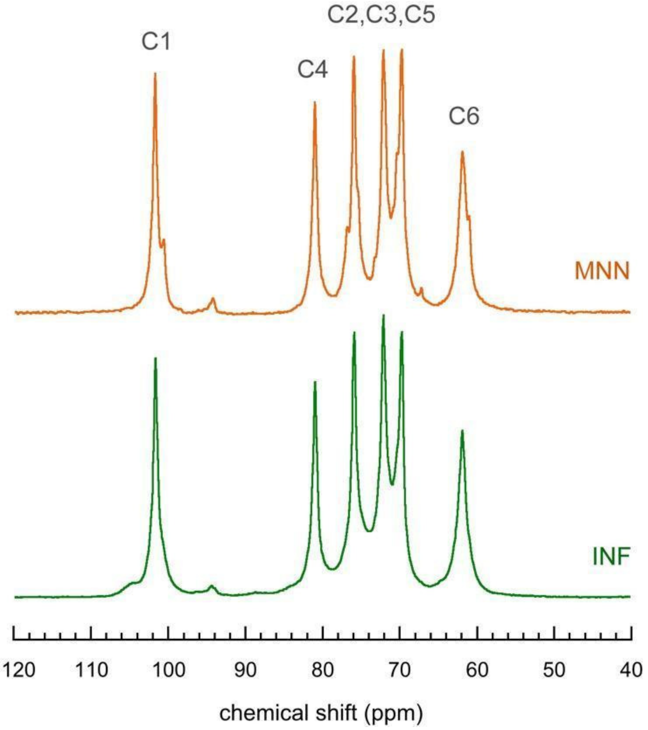

Figure S3. CP/MAS ^{13}C-NMR spectra of ivory nut flour (INF) and recrystallized mannans (MNN). The main peaks correspond to those of mannan I (Marchessault et al., 1990).

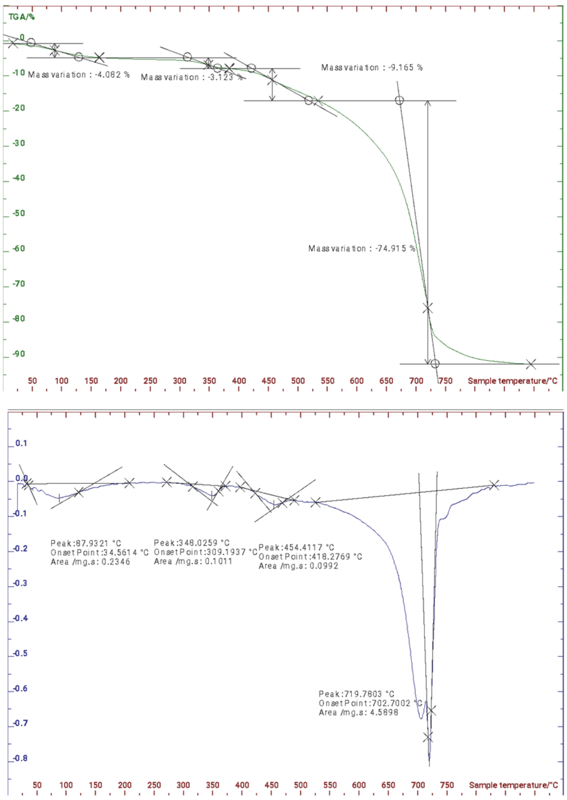

Figure S4. Figure S4. TG (left y-axis) and DTG (right y-axis) curves for ENC upon heating up to 900 ºC. Exo up.
